# Ultraelastic Yarns from Curcumin‐Assisted ELD toward Wearable Human–Machine Interface Textiles

**DOI:** 10.1002/advs.202002009

**Published:** 2020-11-03

**Authors:** Chuang Zhu, Ruohao Li, Xue Chen, Evelyn Chalmers, Xiaoteng Liu, Yuqi Wang, Ben Bin Xu, Xuqing Liu

**Affiliations:** ^1^ Department of Materials, School of Natural Sciences University of Manchester Manchester M13 9PL UK; ^2^ School of Science, Technology, Engineering and Mathematics University of Washington Bothell WA 98011 USA; ^3^ Department of Mechanical and Construction Engineering Faculty of Engineering and Environment Northumbria University Newcastle upon Tyne NE1 8ST UK

**Keywords:** curcumin, electroless deposition, human–machine interfaces, textiles, wearable electronics

## Abstract

Intelligent human–machine interfaces (HMIs) integrated wearable electronics are essential to promote the Internet of Things (IoT). Herein, a curcumin‐assisted electroless deposition technology is developed for the first time to achieve stretchable strain sensing yarns (SSSYs) with high conductivity (0.2 Ω cm^−1^) and ultralight weight (1.5 mg cm^−1^). The isotropically deposited structural yarns can bear high uniaxial elongation (>>1100%) and still retain low resistivity after 5000 continuous stretching–releasing cycles under 50% strain. Apart from the high flexibility enabled by helical loaded structure, a precise strain sensing function can be facilitated under external forces with metal‐coated conductive layers. Based on the mechanics analysis, the strain sensing responses are scaled with the dependences on structural variables and show good agreements with the experimental results. The application of interfacial enhanced yarns as wearable logic HMIs to remotely control the robotic hand and manipulate the color switching of light on the basis of gesture recognition is demonstrated. It is hoped that the SSSYs strategy can shed an extra light in future HMIs development and incoming IoT and artificial intelligence technologies.

## Graphical abstract

An ultralight human–machine interface with excellent stretchability and conductivity is manufactured in this report. Curcumin, a textile dye, is used to modify core‐spun elastic yarns and anchor catalysts for the following metal coating. As‐made stretchable strain sensing yarns can be integrated with cotton gloves via Ecoflex encapsulation for applications in human–machine interfaces and Internet of Things.

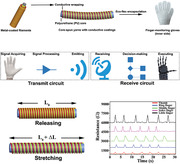

## 1 Introduction

Human–machine interfaces (HMIs), a frontier technology in electronic systems,^[^ ^1^, ^2^, ^3^, ^4^, ^5^, ^6^, ^7^, ^8^ ^]^ have great potentials in developing personal portable electronics and the Internet of Things (IoT) with high integrity and conformal fashion.^[^ ^9^, ^10^, ^11^, ^12^ ^]^ However, there are considerable gaps to achieve highly flexible units that can conformally fit to the curved substrate for gesture recognition and pressure sensing. The recent development of flexible and stretchable sensors has opened a new window to achieve high‐performance wearable HMIs.^[^ ^13^, ^14^, ^15^, ^16^, ^17^, ^18^, ^19^, ^20^ ^]^ Gong et al. developed a stretchable strain sensor by doping polyaniline (PANI) microparticles into gold nanowires (AuNWs), which could be integrated with wireless circuitry to control robotic arms remotely.^[^ ^21^ ^]^ Cao et al. utilized carbon nanotubes (CNTs) and screen printing technique to fabricate a patterned textile electrode^[^ ^22^ ^]^ with good washability and self‐powered touching/gesture sensing functions for the wireless smart home control. Jung et al. synthesized wearable porous pressure‐sensitive rubbers (PPSRs)^[^ ^23^ ^]^ and used the PPSRs‐based HMIs to program the motion of a robot. Despite above attempts on realizing flexible sensing via structural design and/or microprocessing, challenges remain to be tackled such as relatively low conductivity, poor stretchability, and heavy devices, which prevent applications in a higher technology readiness level.

The general methods to achieve flexible sensors with excellent conductivity and durability at low cost are to deposit metals, such as Ni and Cu, on substrates such as textiles,^[^ ^24^, ^25^, ^26^ ^]^ plastic films,^[^ ^27^, ^28^, ^29^ ^]^ fluorine rubbers,^[^ ^30^, ^31^ ^]^ and poly(dimethylsiloxane) (PDMS).^[^ ^32^, ^33^ ^]^ The exercised metallization strategies including sputtering,^[^ ^34^ ^]^ electrochemical deposition,^[^ ^35^ ^]^ and chemical vapor deposition^[^ ^36^ ^]^ to physically or chemically plate metal thin films on flexible substrates have been studied. Compared with those methods, electroless deposition (ELD)^[^ ^37^, ^38^ ^]^ holds promising potentials at a scale‐up level for its cost‐effective nature—no need for expensive instruments and user‐friendly operation conditions, as it can be performed at ambient environment. Polymer‐assisted metal deposition (PAMD) has been regarded as a good complement to ELD,^[^ ^39^, ^40^ ^]^ where a full‐solution process offers a desired anchoring layer to create flexible conductors with enhanced mechanical and electrical features. Zheng and co‐workers previously practiced the above concept by using poly[2‐(methacryloyloxy)ethyl trimethylammonium chloride] brushes to graft onto the fiber surface and immobilize [PdCI_4_]^2−^ species for site‐selective ELD of Cu and Ni on textiles.^[^ ^41^ ^]^ The resulted yarns had conductivity values from 0.28 to 1 S cm^−1^ while showing a high sensitivity to strain changes. Some other approaches offered alternatives to deal with these issues via doping dopamine and tannic acid to facilitate highly conductive and durable textiles.^[^ ^42^, ^43^, ^44^ ^]^ Nevertheless, it is less feasible in practical applications because the dopamine is expensive and tannic acid is unstable in aqueous solution during long‐term storage due to the macromolecular aggregation and self‐precipitation. The attempts are still ongoing for achieving high‐quality and ultralight textile‐based sensors for wearable HMIs.

In this paper, we describe a nature‐inspired strategy to coat a thin metal layer on the elastic core‐spun yarns through solution‐process ELD, to achieve stretchable strain sensing yarns (SSSYs). The key‐enabling feature of this strategy is to introduce curcumin, a natural dye to color textiles in the traditional textile dying process, as the polymer interface to adhere to elastic fibers via *π*–*π* stacking and also capture catalysts through cation–*π* interaction for the subsequent ELD. The curcumin‐assisted ELD offers isotropic metallization to helical nylon strands in the outer layer of yarns to fulfill the electronic conductivity. The obtained helical bangled fiber with sheath–core structure contains the metal‐coated wrapping helices that can respond to small stretching and bending stresses with significant changes in conductivity. We demonstrate the real‐time wireless control of robotic hand by integrating the sensors into a smart glove with signal processing circuit. Moreover, the smart glove system shows potential applications in IoT by programming the color change of light with gesture controls.

## 2 Results and Discussion

**Figure 1:**
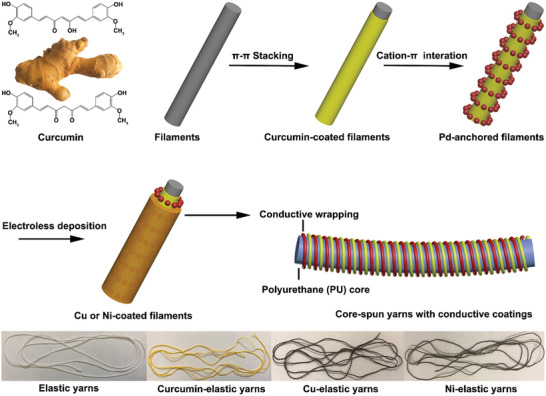
The illustration of fabrication process of SSSYs and observations of elastic yarns in each different state (bottom).

The structure design of SSSYs is illustrated in **Figure** **1**. In brief, curcumin was applied to promote the adhesion on the fiber surface via intermolecular hydrogen bonds and *π*–*π* stacking to nest catalysts, leading to the successful ELD metallization of Cu or Ni on the surface of wrapping filaments at ambient conditions. These as‐prepared SSSYs with conductive outer layers and the elastic core present superior flexibility to be conformally attached to the inner side of gloves via Ecoflex filtration in room temperature.

**Figure 2:**
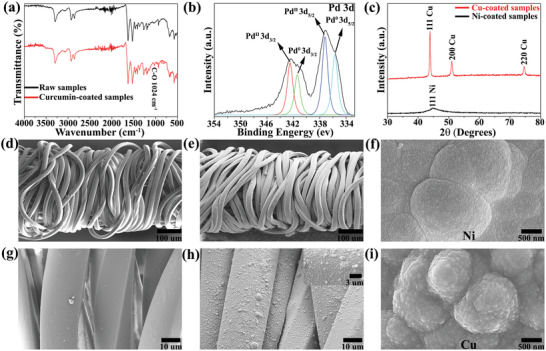
a) The transmittance FTIR spectrum of the raw elastic yarn (black) and the curcumin‐coated core‐spun yarn (red). b) The XPS spectrum of Pd 3d for the Pd/curcumin sample. c) The XRD spectrum of Ni‐coated samples (black) and Cu‐coated yarns (red). d–i) SEM images of the pristine yarn, the copper‐coated yarn (30 min ELD), Ni coatings (60 min ELD) at 2.5 keV, the pristine yarn in high magnification, the copper‐coated yarn in high magnification (the inset is the magnified image), and Cu coatings (60 min ELD) at the low‐voltage mode, respectively.

The surface chemical and physical properties of elastic yarns were assessed for each process to reveal the molecular interplays. The Fourier transform infrared (FTIR) spectroscopy technique was applied to confirm the successful coating of curcumin on fibers. As shown in **Figure** **2**a, compared with the FTIR spectrum of raw samples and pure curcumin from ref. ^[^ ^45^ ^]^, the FTIR spectrum of curcumin‐coated samples shows an intense band at 1024 cm^−1^ attributed to stretching vibrations of C—O groups in curcumin. X‐ray photoelectron spectroscopy (XPS) was used to characterize the catalytic fibers to study the surface chemical composition, where a uniform distribution of elements was captured in Pd/curcumin yarns (Figure S1, Supporting Information). As shown in Figure 2b, the complex spectrum of Pd 3d electrons for a palladium loaded specimen is decomposed into two spin–orbital doublets, which are accounted for two electronic states of palladium: Pd (0) (binding energy 335.7 and 341.4 eV) and Pd (II) (337.2 and 342.5 eV). This indicates both reduction and chelation of palladium cations are accumulated in the curcumin‐modified surface.^[^ ^46^ ^]^ The stronger Pd(II) doublet peaks demonstrate that more palladium ions were chelated by curcumin, which would enhance the catalytic activity in ELD because the catalytic performance of reduced Pd atoms is very poor, especially when metal particles are large and nonuniform. We noticed that, compared with the reported values,^[^ ^47^, ^48^, ^49^, ^50^ ^]^ Pd (II) peaks are negatively shifted by 1 eV, which could be attributed to that the curcumin layer gives a bigger electron‐donating effect toward palladium cations, which may also facilitate the catalytic activity through altering their adsorption properties toward poisonous species such as CO.

After the catalytic activation, ELD‐assisted metallization was performed to coat conformal layers of nanoparticles on the fiber surface. The morphological observation of coated layers from scanning electron microscope (SEM) suggests a significant change of surface from smooth state for the raw core‐spun yarn (Figure 2d–g), to homogeneous and conformal Cu layers for the Cu‐coated yarn (Figure 2e–h). After Ni ELD, the nickel coating on the surface of elastic yarns is also very uniform, continuous, and dense (Figure S2, Supporting Information). X‐ray diffraction (XRD) was performed to ascertain the crystalline structure and size of metal deposits. As shown in Figure 2c, the XRD pattern of Ni‐deposited yarns shows a broad diffraction peak at the 2*θ* value of 44.90 corresponding to the (111) plane of face‐centered cubic (FCC) phase nickel (JCPDS Card No. 45‐1027), which implies good conductivity of as‐prepared conductive yarns. For the XRD pattern of Cu‐coated samples, three distinct characteristic peaks at 2*θ* values of 43.90, 51.00, and 74.70 are assigned to the Cu (111), Cu (200), and Cu (220) planes, respectively. This diffraction pattern of Cu‐coated yarns matches exactly with the standard pattern of cooper (JCPDS File No. 04‐0836), and no diffraction peaks corresponding to copper oxide are observed, suggesting the excellent stability of metal coating. According to the Scherrer equation, the average sizes of nickel and copper deposits were calculated to be 17.3 and 31.3 nm, respectively. Based on scale bars in SEM images of metal‐coated surfaces under a low‐voltage SEM at 2.5 keV (Figure 2f–i), it can be clearly seen that the sizes of grains are around 15–20 nm for Ni coating layers (*n* = 5) and 20–40 nm for Cu films (*n* = 5), which are in reasonable agreements with the theoretical values.

**Figure 3:**
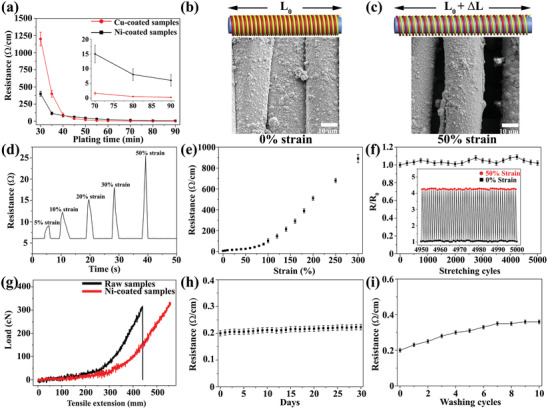
a) The conductivity results of Ni (black) or Cu (red)‐coated yarns at different ELD durations (*n* = 5). SEM images and the scheme illustrations of nickel‐coated yarns at b) 0% strain and c) 50% strain. d) The resistance changes of conductive yarns when stretching and releasing at different strains. e) The resistance changes of as‐made nickel‐based yarns when continuously stretching to 300% elongation (*n* = 5). f) Resistance stability of sensor materials under repetitive stretching (50% strain) and relaxing (0% strain) cycles (the change in resistance is defined as: *R*/*R*_0_) (*n* = 5). g) The tensile test of raw elastic yarns and Ni‐coated stretchable yarns (the original sample length is 45 mm). The durability test of Cu‐coated samples including h) air stability (*n* = 5) and i) washing fastness (*n* = 5).

Compared with insulated textiles, the as‐prepared SSSYs are electrically conductive. The electrical surface resistance of metal‐coated core‐spun yarns decreased with extending depositing time due to more metal nanoparticles deposited on fiber surface, as shown in **Figure** **3**a. The resistance of nickel‐coated samples and copper‐plated yarns can reach as low as 5 and 0.2 Ω cm^−1^ at 90 min, respectively. The resistance of copper‐coated yarns is much lower than that of nickel‐deposited samples because of the higher intrinsic conductivity of bulk Cu. Upon stretching, the helical structure wrapped yarns elongated in the longitudinal direction, where the detachment of the adjacent metal‐deposited nylon yarns occurred, leading to an increase in resistance of SSSYs due to the loss of direct contacted area, as shown in Figure 3b,c for the nickel‐coated nylon warping on the PU core under strain from 0% to 50%. This strain‐dependent conductivity is further investigated on Ni‐coated elastic yarns (Figure 3d). By applying 5% elongation, we found that the electrical resistance of as‐prepared yarns witnessed a small increase from 6 to 9 Ω and fully resumed from 9 to 6 Ω once the external load was withdrawn because the nickel‐coated nylon shell was brought into contact. The elasticity‐enabled reversibility was then examined at higher elongation strains, e.g., 10%, 20%, 30%, and 50%, where the resistance of conductive yarns increased from 6 to 12.3, 15.2, 18.1, and 25.5 Ω, respectively, and recovered to the original values after unloading tensile forces.

In order to understand the electrical resistance evolution for the ELD‐coated yarn at high elongation, we measured the strain–resistance response of the Ni‐coated yarn under uniaxially stretching till it breaks. As shown in Figure 3e, the resistance of the nickel‐based elastic yarns increased gradually to 25.5 Ω at 50% strain. When further increasing elongation, the resistance of as‐made conductive fibers showed a dramatic increase due to the cracking and peeling off of nickel coating layers (Figure S3, Supporting Information), which could lead to the failure of local electrical network. We also found that the tensile performance of composite yarns is enhanced (Figure 3g), where the Ni‐coated elastic yarns show much higher tolerable strain (>>1100%) (the upper limit of the tensile machine is 550 mm), compared with the bare yarns. The Cu‐coated elastic yarns showed the same tensile property as Ni‐deposited composite yarns. This is due to the homogeneous metal coating on nylon fiber surface, decreasing the frictional force between fibers. We next assessed the durability of metal‐coated yarn by cyclic stretching at a fixed strain of 50%. A robust strain–resistance relationship was discovered for Ni‐coated yarns after long‐term stretching–releasing cycles (up to 5000, Figure 3f). Moreover, only a small increase in resistance was found for Cu‐based elastic yarns after being exposed to the natural environment for 30 d (Figure 3h) or several washing cycles (Figure 3i), which shows the potential applications of Cu‐coated yarns in harsh environment.

**Figure 4:**
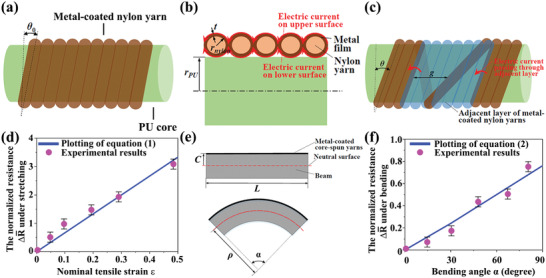
Deformation mechanics analysis of the metal‐coated core‐spun yarns: structural illustrations with a) side view without deformation, b) cross‐sectional view without deformation, and c) detached nylon windings under tension. d) The comparison of theoretical prediction with experimental results for SSSY under tension (*n* = 3). e) Simplified beam model for the deformation of SSSY attached in smart glove system (a straight beam when relaxing and a deformed beam when bending), *ρ* is the radius of the deformed beam. f) The comparison of analytical prediction with experimental values for bending angle–resistance responses of Ni‐coated core‐spun yarns (*n* = 3).

To further investigate the working mechanism of SSSYs, we conducted the theoretical study on resistance responses of metal‐coated core‐spun yarns under releasing, uniaxial stretching and bending. As shown in **Figure** **4**a, SSSYs are decomposed as: PU core fiber, nylon yarns bangling around the core, and metal (Cu or Ni) film conformally deposited on the surface of nylon helices. At the initial state (0% strain), the adjacent metal‐coated nylon rims are in contact with each other (Figure 4b). Under stretching, the winding angle (*θ*) and *N*_detach_ detached windings appear with an average gap of *g*, as illustrated in Figure 4c. The full developments for scaling are given in the Supporting Information. Generally, the winding angle *θ*, the average gap *g* of the detached nylon windings, and the resistance *R*_detach_ will increase during the stretching. Therefore, the normalized resistance change, Δ$\bar{R}$, can be expressed as a function of tensile strain *ε*, *θ*(*ε*) (*θ* as a function of *ε*), *g*(*ε*) (*g* as a function of *ε*), and *R*_detach_(*ε*) (*R*_detach_ as a function of *ε*). It should be noted that *θ* changes very limited for the SSSYs when the applied strain is less than 50% (see Figure 3b,c). Thus, we can obtain the following scaling equation for the yarn under stretching

(1) $$\begin{array}{ccc} \Delta \bar{R} & = & \left(\left(\frac{R_{d}^{\left(0\right)}}{\frac{\rho_{\mathrm{metal}}}{4t}\cdot \frac{r_{\mathrm{nylon}}}{r_{\mathrm{PU}}}}-\frac{1}{1-\nu \varepsilon }\right)\frac{r_{\mathrm{nylon}}/\cos\theta_{0}}{g_{0}}\right)\\ & & \times \;\varepsilon +\left(\frac{1}{1-\nu \varepsilon }-1\right) \end{array}$$

where *ρ*_metal_ and *t* are the electrical resistivity and thickness of metal film (Ni or Cu), respectively, *ν* is the Poisson's ratio of PU, *r*_nylon_ is the radius of nylon yarn, *r*_PU_ is the radius of PU core (see Figure 4b), *θ*_0_ is the winding angle at initial state, *g*_0_ is the assumed constant gap of detaching winding, and $R_{d}^{\left(0\right)}$ is the assumed constant resistance of the detached winding. In addition to the tensile strain *ε*, Δ$\bar{R}$ is found with dependencies on other two dimensionless parameters: relative resistance increase of one detached winding Δ${\bar{R}}_{d}$ ( $=\frac{R_{d}^{\left(0\right)}}{\frac{\rho_{\mathrm{metal}}}{4t}\frac{r_{\mathrm{nylon}}}{r_{PU}}}\;$) and the relative detached gap $\bar{g}$ ( $=\frac{g_{0}}{r_{\mathrm{nylon}}/\cos\theta_{0}}\;$). We can obtain *r*_PU_ = 155 µm, *r*_nylon_ = 11 µm from Figure 2 and *θ*_0_ = 3° and *g*_0_ = 8 µm from Figure 3, and take *t* = 17.3 nm, *ρ*_Ni_ = 6.99 × 10^−8^ Ωm, *ν* = 0.5 and $R_{d}^{\left(0\right)}=\;0.41\;\Omega$. Given by those data, this scaling relationship under stretching can be calculated as shown in Figure 4d, where the theoretical results are in good agreement with the experimental results of Ni‐coated core‐spun yarns.

From the perspective of bending in the following HMI system, a simplified model is created by assuming structural fiber as a beam (substrate) with metal‐coated core‐spun yarn attached to its top surface (Figure 4e). Under bending, we then can obtain the scaling equation for bending as follows (please see full derivations in the Supporting Information)

(2) $$\begin{array}{ccc} \Delta \bar{R} & = & \left(\left(\frac{R_{d}^{\left(0\right)}}{\frac{\rho_{\mathrm{metal}}}{4t}\cdot \frac{r_{\mathrm{nylon}}}{r_{\mathrm{PU}}}}-\frac{1}{1-\nu \cdot \frac{c}{L}\cdot \alpha }\right)\frac{r_{\mathrm{nylon}}/\cos\theta_{0}}{g_{0}}\frac{c}{L}\right)\\ & & \times \;\alpha +\left(\frac{1}{1-\nu \cdot \frac{c}{L}\cdot \alpha }-1\right) \end{array}$$

where *L* is the initial length of the beam, *c* is the distance between the top surface of the beam and its neutral surface (i.e., the surface at which the tensile strain is 0%), and *α* is the bending angle of the beam (see Figure 4e). With $\frac{c}{L}=\;0.07$ (index finger as the substrate beam) and other parameter values given in the above discussion, Equation (2) is plotted in Figure 4f, which shows that Δ$\bar{R}$ under bending is nearly proportional to *α*. The experimental results of Ni‐based composite yarns under bending also highly agree with the theoretical model.

**Figure 5:**
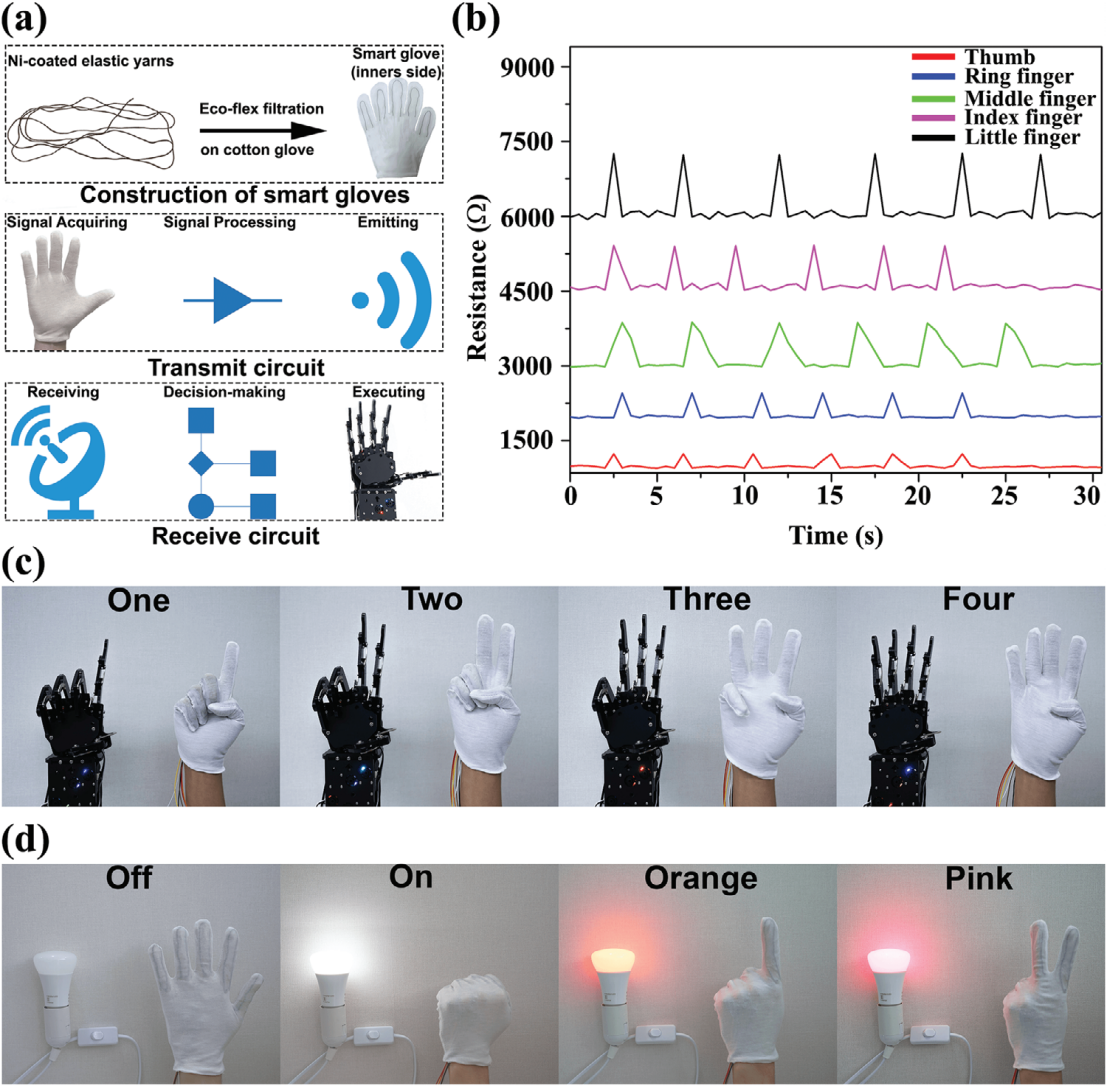
a) Schematic diagram of the human–machine remote control system. b) The resistance values of five fingers under different strains. Applications c) to control the robotic hand and d) to control the color of light by using hand gestures.

On the basis of the excellent strain–resistance response discussed above, our as‐fabricated conductive core‐spun yarns were demonstrated to monitor the motion of human fingers and realize the remote control of the robotic hand. In detail, Ecoflex was used to adhere Ni‐based sensor materials to the inner side of cotton gloves by mixing A and B solutions with weight ratio 1:1. Importantly, the Ecoflex encapsulation layer, as a skin safe product, not only prevents the direct contact from nickel to human skin but also protects the nickel coating from oxidization. The weights of the smart glove and the built‐in sensor materials are only 8.5 and 0.1g, respectively, because of the lightweight nature of fiber assemblies. With the as‐fabricated ultralight smart glove, the overall control system consisting of transmit circuit and receive circuit can be designed, as depicted in **Figure** **5**a.

The algorithm in this system uses the resistance changes of each yarn‐based sensor in the finger part of the glove as signal inputs, analyzes ascending/descending resistance and converts resistance value into servo angles. Each finger of the robotic hand has a servo connection and thus, this algorithm of smart gloves produces a real‐time reaction, i.e., the robotic hand performs the same gesture when the user operates the smart glove. Smart gloves, analog‐to‐digital converter (ADC), digital signal processing (DSP), wireless module (HC‐12), digital‐to‐analog converter (DAC), robotic hand servos, and the robotic palm are major components of the designed system. In the transmit circuit (Figure S4, Supporting Information), when the bending/releasing motions of human fingers were perceived by yarn‐based sensors, quantitative pulses were generated because the attached yarns were stretched/released, leading to less/more contact area between wrapping nylon and increased/decreased resistance. The microscope images of composite yarns confirmed that bending fingers caused less Ni‐coated nylon brought into contact because such yarns experienced elongation (Figure S5, Supporting Information). Five ADCs then converted these analog inputs into digital outputs by Arduino programming in the Arduino board. Afterward, DSP extracted outliers from the interval between the highest threshold and the lowest threshold (Table S1, Supporting Information) and the HC‐12 wireless communication module finally sent these digital signals to the receive circuit.

Importantly, in order to provide more signals for DSP, a wide interval needs to be created and the interval for each finger cannot be overlapped. To achieve this, high‐resistance sensor materials were manufactured by controlling the ELD time and from our previous report, the high resistance metal‐based yarns exhibit superior flexibility and durability because of the multilayers structure of the metal film. Therefore, five SSSYs from 10 to 30 min ELD were obtained. As shown in Figure 5b, nickel‐coated yarns experienced a large increase when being stretched (≈2.5% strain) and reversed to their original values when relaxing. In the receive circuit (Figure S6, Supporting Information), all five digital resistance values were received by another HC‐12 wireless communication module and these data would be then converted into five digital servo angle signals. In the last step, five DACs converted digital servo signals into analog servos for different robot fingers. Thus, the robotic hand moved its specified finger to a determined position based on the individual analog angle input. As shown in Figure 5c, the operator's real hand gestures including different numbers (1, 2, 3, and 4) were accurately reflected by the robotic hands. The related video can be found in Movie S1 (Supporting Information).

Apart from controlling the robotic palm, as‐prepared smart gloves also have potential applications in IoT by using If This, Then That (IFTTT) protocol through WIFI modules. To realize this, the Philips Hue lightbulb which supports the IFTTT protocol was used in the IoT system. The core idea of IoT is to assign unique identifiers (UIDs) to the interrelated computing devices, machines, objects, animals, or people and transfer data over a network with automatic control. The IFTTT protocol is a platform which triggers actions on the hardware if the received signals reach the threshold. From Figure S7 (Supporting Information), the WIFI module enhanced Arduino board collected resistance values when bending/releasing fingers. Subsequently, the built‐in DSP in the WIFI module boosted Arduino board would analyze the value of resistance based on designed algorithm above and then posted a trigger to IFTTT. In the final step, the light executed the action based on different triggers. In Figure 5d, we coded the gestures to control the bulb such as showing palm to turn off the light, closing palm or “fist” to turn off the light, hand signal of “one” for orange color, and hand signal of “two” for pink light. More triggers based on different finger combinations were designed and demonstrated in Movie S2 (Supporting Information).

## 3 Conclusion

In summary, highly reliable and stretchable strain sensors based on metal‐coated core‐spun yarns were developed via a low‐cost, facile, and scalable method. The unique structure of covered elastic yarns endowed sensor materials with excellent stretchability (>>1100%) and high durability during 5000 continuous stretching–releasing cycles under 50% strain without obvious damage. The superb cyclic performance should be assigned to the predecorated curcumin interface, which significantly increases the adhesion between metal nanoparticles and flexible substrates. The highly sensitive and durable nature of as‐prepared conductive yarns equipped them as promising wearable strain sensors with excellent integration and adaptivity. Moreover, the theoretical model study was conducted to explore the working mechanism of SSSYs. By integrating these SSSY sensors with commercial cotton gloves, the real‐time wireless control of the robotic palm could be successfully implemented by using the signal processing circuit. More interestingly, the concept of smart glove was advanced into the application in IoT to manipulate the colors of light based on a gesture controlling mechanism. We expected that this approach would open a new window in designing wearable devices for applications in HMIs and IoT such as smart home controlling, healthcare, and industrial automation.

## 4 Experimental Section

##### Materials

Curcumin, ammonium tetrachloropalladate (II) [(NH_4_)_2_PdCI_4_], ethanol absolute, and all other chemicals were purchased from Sigma‐Aldrich. Core‐spun elastic yarns were obtained from the Dye House at the University of Manchester. Cotton glove, robotic hand, Philips Hue lightbulb, and signal processing units were purchased from the market. Each substrate was ultrasonically cleaned in acetone and distilled (DI) water for 30 min, respectively and dried with a N_2_ gas stream.

##### Polymer Interface Design

Curcumin was dissolved in ethanol absolute to prepare a curcumin solution (5 g L^−1^). Cleaned samples were then immersed into the solution for 30 min and the curcumin‐coated samples were rinsed with DI water for several times, followed by N_2_ drying.

##### Electroless Deposition

Catalyst species were captured by dipping curcumin‐grafted samples into a 5 × 10^−3^ m (NH_4_)_2_PdCI_4_ aqueous solution and then placed in a dark environment for 15 min. DI water rinsing was applied to remove the physical absorption of catalysts. The Ni ELD was conducted in a plating bath containing 4:1 mixture of solution A and B at ambient environment. Solution A made of 10 g L^−1^ lactic acid, 20 g L^−1^ sodium citrate, and 40 g L^−1^ nickel sulfate hexahydrate in DI water was prepared in advance. Solution B containing 1 g L^−1^ dimethylamine borane (DMAB) in DI water was freshly prepared. The pH of mixed solutions was adjusted to ≈8 before immersing catalytic samples. The Cu ELD was conducted in plating bath composed of 1:1 mixture of the Cu stock solution and freshly prepared 9.5 mL L^−1^ HCHO in DI water. The copper stock containing 12 g L^−1^ NaOH, 13 g L^−1^ CuSO_4_·5H_2_O, and 29 g L^−1^ KNaC_4_H_4_O_6_·4H_2_O in DI water was prepared in advance. After ELD, all samples were washed for several times and subsequently dried with compressed air.

##### Characterization

The modification of curcumin on elastic yarns was tested by Fourier transform infrared spectroscopy (NICOLET 5700 FTIR). The surface composition of catalyst‐immobilized samples was analyzed by XPS Near Ambient Pressure. The surface morphology of the samples was investigated by scanning electron microscope (ZEISS Ultra‐55). The size and the shape of the unit cell for metallic particles on the sample surface were characterized by X‐ray diffraction (PANalytical X'Pert Pro X'Celerator diffractometer). The tensile property of metal‐coated yarns was tested by tensile machine Instron 3345. A two‐point probe method with a Keithley 2000 Multimeter was used to measure the resistance of metal‐coated stretchable yarns.

##### Statistical Analysis

Statistical analysis was compiled on the means of the data obtained from at least three independent experiments using Origin software. All values were expressed as the mean ± standard deviation (SD) of individual sample. The sample size (*n*) numbers for each experiment were indicated in the figure legends.

## Conflict of Interest

The authors declare no conflict of interest.

## Supporting information

Supporting Information

Supplemental Movie 1

Supplemental Movie 2

## References

[1] K. Sim , Z. Rao , Z. Zou , F. Ershad , J. Lei , A. Thukral , J. Chen , Q.‐A. Huang , J. Xiao , C. Yu , Sci. Adv. 2019, 5, eaav9653. 31414044 10.1126/sciadv.aav9653 PMC6677552

[2] O. Y. Kweon , S. J. Lee , J. H. Oh , NPG Asia Mater. 2018, 10, 540.

[3] Y. J. Tan , H. Godaba , G. Chen , S. T. M. Tan , G. Wan , G. Li , P. M. Lee , Y. Cai , S. Li , R. F. Shepherd , J. S. Ho , B. C. K. Tee , Nat. Mater. 2020, 19, 182. 31844282 10.1038/s41563-019-0548-4

[4] F. Yi , L. Lin , S. Niu , J. Yang , W. Wu , S. Wang , Q. Liao , Y. Zhang , Z. L. Wang , Adv. Funct. Mater. 2014, 24, 7488.

[5] C. Tawk , M. in het Panhuis , G. M. Spinks , G. Alici , Adv. Intell. Syst. 2019, 1, 1900002.

[6] E. Roh , B.‐U. Hwang , D. Kim , B.‐Y. Kim , N.‐E. Lee , ACS Nano 2015, 9, 6252. 25869253 10.1021/acsnano.5b01613

[7] W. Deng , T. Yang , L. Jin , C. Yan , H. Huang , X. Chu , Z. Wang , D. Xiong , G. Tian , Y. Gao , H. Zhang , W. Yang , Nano Energy 2019, 55, 516.

[8] Z. Li , Y. Ma , L. Wang , X. Du , S. Zhu , X. Zhang , L. Qu , M. Tian , ACS Appl. Mater. Interfaces 2019, 11, 46278. 31713408 10.1021/acsami.9b19078

[9] L. Zhang , J. He , Y. Liao , X. Zeng , N. Qiu , Y. Liang , P. Xiao , T. Chen , J. Mater. Chem. A 2019, 7, 26631.

[10] Y. Liu , J. J. S. Norton , R. Qazi , Z. Zou , K. R. Ammann , H. Liu , L. Yan , P. L. Tran , K.‐I. Jang , J. W. Lee , D. Zhang , K. A. Kilian , S. H. Jung , T. Bretl , J. Xiao , M. J. Slepian , Y. Huang , J.‐W. Jeong , J. A. Rogers , Sci. Adv. 2016, 2, e1601185. 28138529 10.1126/sciadv.1601185 PMC5262452

[11] Y.‐C. Huang , Y. Liu , C. Ma , H.‐C. Cheng , Q. He , H. Wu , C. Wang , C.‐Y. Lin , Y. Huang , X. Duan , Nat. Electron. 2020, 3, 59.

[12] Z. F. Liu , S. Fang , F. A. Moura , J. N. Ding , N. Jiang , J. Di , M. Zhang , X. Lepró , D. S. Galvão , C. S. Haines , N. Y. Yuan , S. G. Yin , D. W. Lee , R. Wang , H. Y. Wang , W. Lv , C. Dong , R. C. Zhang , M. J. Chen , Q. Yin , Y. T. Chong , R. Zhang , X. Wang , M. D. Lima , R. Ovalle‐Robles , D. Qian , H. Lu , R. H. Baughman , Science 2015, 349, 400. 26206929 10.1126/science.aaa7952

[13] Z. Liu , D. Qi , G. Hu , H. Wang , Y. Jiang , G. Chen , Y. Luo , X. J. Loh , B. Liedberg , X. Chen , Adv. Mater. 2018, 30, 1704229. 10.1002/adma.201704229 29226515

[14] G. Cheng , H. Zheng , F. Yang , L. Zhao , M. Zheng , J. Yang , H. Qin , Z. Du , Z. L. Wang , Nano Energy 2018, 44, 208.

[15] L. Dhakar , P. Pitchappa , F. E. H. Tay , C. Lee , Nano Energy 2016, 19, 532.

[16] J. Chen , G. Zhu , J. Yang , Q. Jing , P. Bai , W. Yang , X. Qi , Y. Su , Z. L. Wang , ACS Nano 2015, 9, 105. 25552331 10.1021/nn506832w

[17] Y. Ai , Z. Lou , S. Chen , D. Chen , Z. M. Wang , K. Jiang , G. Shen , Nano Energy 2017, 35, 121.

[18] X. Hu , M. Tian , T. Xu , X. Sun , B. Sun , C. Sun , X. Liu , X. Zhang , L. Qu , ACS Nano 2020, 14, 559. 31855404 10.1021/acsnano.9b06899

[19] F. Sun , M. Tian , X. Sun , T. Xu , X. Liu , S. Zhu , X. Zhang , L. Qu , Nano Lett. 2019, 19, 6592. 31434486 10.1021/acs.nanolett.9b02862

[20] Z. Wang , Y. Huang , J. Sun , Y. Huang , H. Hu , R. Jiang , W. Gai , G. Li , C. Zhi , ACS Appl. Mater. Interfaces 2016, 8, 24837. 27558025 10.1021/acsami.6b08207

[21] S. Gong , D. T. H. Lai , Y. Wang , L. W. Yap , K. J. Si , Q. Shi , N. N. Jason , T. Sridhar , H. Uddin , W. Cheng , ACS Appl. Mater. Interfaces 2015, 7, 19700. 26301770 10.1021/acsami.5b05001

[22] R. Cao , X. Pu , X. Du , W. Yang , J. Wang , H. Guo , S. Zhao , Z. Yuan , C. Zhang , C. Li , Z. L. Wang , ACS Nano 2018, 12, 5190. 29771494 10.1021/acsnano.8b02477

[23] S. Jung , J. H. Kim , J. Kim , S. Choi , J. Lee , I. Park , T. Hyeon , D.‐H. Kim , Adv. Mater. 2014, 26, 4825. 24827418 10.1002/adma.201401364

[24] D. Wang , Y. Zhang , X. Lu , Z. Ma , C. Xie , Z. Zheng , Chem. Soc. Rev. 2018, 47, 4611. 29722373 10.1039/c7cs00192d

[25] B. K. Little , Y. Li , V. Cammarata , R. Broughton , G. Mills , ACS Appl. Mater. Interfaces 2011, 3, 1965. 21574628 10.1021/am200193c

[26] X. Lin , M. Wu , L. Zhang , D. Wang , ACS Appl. Electron. Mater. 2019, 1, 397.

[27] R. Guo , Y. Yu , Z. Xie , X. Liu , X. Zhou , Y. Gao , Z. Liu , F. Zhou , Y. Yang , Z. Zheng , Adv. Mater. 2013, 25, 3343. 23670964 10.1002/adma.201301184

[28] S. Liang , Y. Li , T. Zhou , J. Yang , X. Zhou , T. Zhu , J. Huang , J. Zhu , D. Zhu , Y. Liu , C. He , J. Zhang , X. Zhou , Adv. Sci. 2017, 4, 1600313. 10.1002/advs.201600313 PMC5323856 28251052

[29] H. Zhang , P. Zhang , H. Zhang , X. Li , L. Lei , L. Chen , Z. Zheng , Y. Yu , ACS Appl. Mater. Interfaces 2018, 10, 28963. 30080380 10.1021/acsami.8b08014

[30] X. Wang , H. Hu , Y. Shen , X. Zhou , Z. Zheng , Adv. Mater. 2011, 23, 3090. 21598315 10.1002/adma.201101120

[31] D. L. Gao , M. S. Zhan , Appl. Surf. Sci. 2009, 255, 4185.

[32] M. S. Miller , G. J. E. Davidson , B. J. Sahli , C. M. Mailloux , T. B. Carmichael , Adv. Mater. 2008, 20, 59.

[33] F.‐T. Zhang , L. Xu , J.‐H. Chen , B. Zhao , X.‐Z. Fu , R. Sun , Q. Chen , C.‐P. Wong , ACS Appl. Mater. Interfaces 2018, 10, 2075. 29253331 10.1021/acsami.7b15726

[34] D. K. Diop , L. Simonot , N. Destouches , G. Abadias , F. Pailloux , P. Guérin , D. Babonneau , Adv. Mater. Interfaces 2015, 2, 1500134.

[35] A. P. Abbott , K. J. McKenzie , Phys. Chem. Chem. Phys. 2006, 8, 4265. 16986069 10.1039/b607329h

[36] Y. Sun , J. A. Rogers , Adv. Mater. 2007, 19, 1897.

[37] L. Liu , Y. Yu , C. Yan , K. Li , Z. Zheng , Nat. Commun. 2015, 6, 7260. 26068809 10.1038/ncomms8260 PMC4490556

[38] Y. You , Z. Yaokang , L. Kan , Y. Casey , Z. Zijian , Small 2015, 11, 3444.25786920

[39] P. Li , Y. Zhang , Z. Zheng , Adv. Mater. 2019, 31, 1902987. 10.1002/adma.201902987 31304644

[40] C. Zhu , Y. Li , X. Liu , Polymers 2018, 10, 573. 10.3390/polym10060573 PMC6404067 30966608

[41] X. Liu , H. Chang , Y. Li , W. T. Huck , Z. Zheng , ACS Appl. Mater. Interfaces 2010, 2, 529. 20356201 10.1021/am900744n

[42] C. Zhu , X. Guan , X. Wang , Y. Li , E. Chalmers , X. Liu , Adv. Mater. Interfaces 2019, 6, 1801547.

[43] C. Zhu , E. Chalmers , L. Chen , Y. Wang , B. B. Xu , Y. Li , X. Liu , Small 2019, 15, 1902440. 10.1002/smll.201902440 31215162

[44] H. Lee , S. M. Dellatore , W. M. Miller , P. B. Messersmith , Science 2007, 318, 426. 17947576 10.1126/science.1147241 PMC2601629

[45] M. M. Yallapu , M. Jaggi , S. C. Chauhan , Colloids Surf., B 2010, 79, 113. 10.1016/j.colsurfb.2010.03.039 20456930

[46] J. Xi , J. Xiao , F. Xiao , Y. Jin , Y. Dong , F. Jing , S. Wang , Sci. Rep. 2016, 6, 21904. 26902657 10.1038/srep21904 PMC4763215

[47] E. Y. Pisarevskaya , V. I. Zolotarevskiy , L. P. Kazanskiy , E. V. Ovsyannikova , N. M. Alpatova , Synth. Met. 2009, 159, 304.

[48] H. Yang , S. Kang , H. Zou , J. Jin , J. Ma , S. Li , RSC Adv. 2016, 6, 90462.

[49] P. Wu , Y. Huang , L. Zhou , Y. Wang , Y. Bu , J. Yao , Electrochim. Acta 2015, 152, 68.

[50] I. Aruna , B. R. Mehta , L. K. Malhotra , S. M. Shivaprasad , J. Appl. Phys. 2008, 104, 064308.

