# Supplementary material for: Ultraelastic Yarns from Curcumin‐Assisted ELD toward Wearable Human–Machine Interface Textiles
Source: Adv Sci (Weinh). 2020 Nov 3;7(23):2002009. doi: 10.1002/advs.202002009 (PMC7709996; doi:10.1002/advs.202002009)
Supplement: Supplementary file 1 — Supporting Information [file ADVS-7-2002009-s001.pdf]

© 2020 Wiley-VCH GmbH

ADVANCED SCIENCE
Open Access

Supporting Information

for *Adv. Sci.,* DOI: 10.1002/advs.202002009

Ultraelastic Yarns from Curcumin-assisted ELD towards Wearable Human-Machine Interface Textiles

Chuang Zhu, Ruohao Li, Xue Chen, Evelyn Chalmers, Xiaoteng, Liu, Yuqi Wang, Ben Bin Xu and Xuqing Liu**

WILEY-VCH

© 2020 Wiley-VCH GmbH

Supporting Information

Ultraelastic yarns from curcumin-assisted ELD towards Wearable human-Machine interface textiles

Chuang Zhu, Ruohao Li, Xue Chen, Evelyn Chalmers, Xiaoteng, Liu, Yuqi Wang, Ben Bin Xu and Xuqing Liu**

Supporting Information

Ultraelastic Yarns from Curcumin-assisted ELD towards Wearable Human-Machine Interface Textiles

Chuang Zhu, Ruohao Li, Xue Chen, Evelyn Chalmers, Xiaoteng, Liu, Yuqi Wang, Ben Bin Xu and Xuqing Liu**

C. Zhu, Y. Q. Wang, E. Chalmers, Dr. X. Q. Liu
Department of Materials, University of Manchester, Manchester, M13 9PL, UK
E-mail: xuqing.liu@manchester.ac.uk

R. H. Li
School of Science, Technology, Engineering and Mathematics, University of Washington, Bothell, WA 98011, USA

X. Chen, X. T. Liu, Dr. B. B. Xu
Department of Mechanical and Construction Engineering, Faculty of Engineering and Environment, Northumbria University, Newcastle upon Tyne, NE1 8ST, UK
E-mail: ben.xu@northumbria.ac.uk

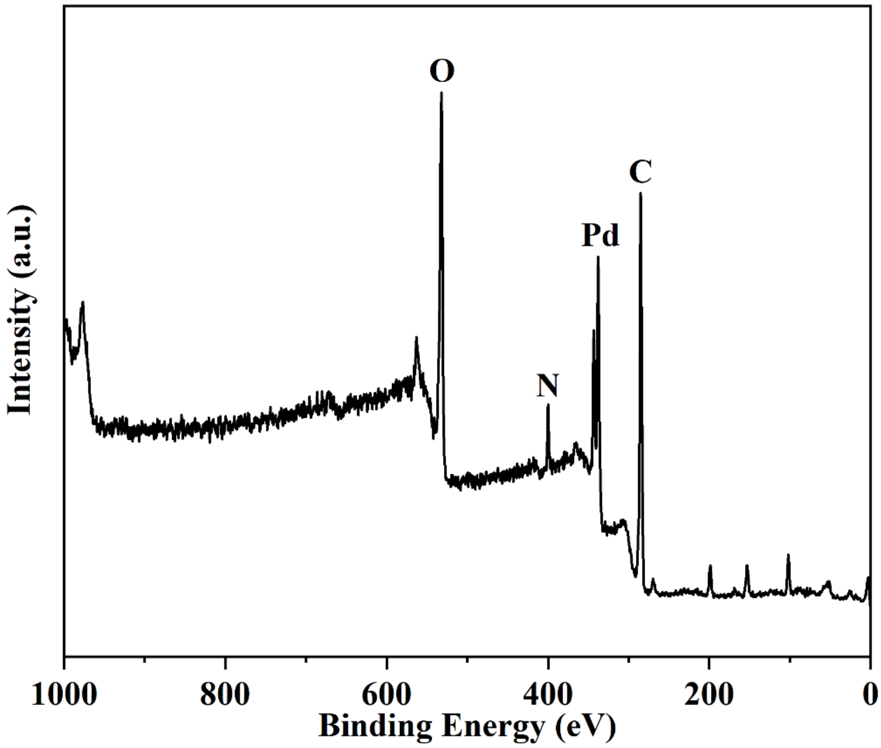

Figure S1. XPS survey spectrum of Pd/Curcumin-elastic yarns.

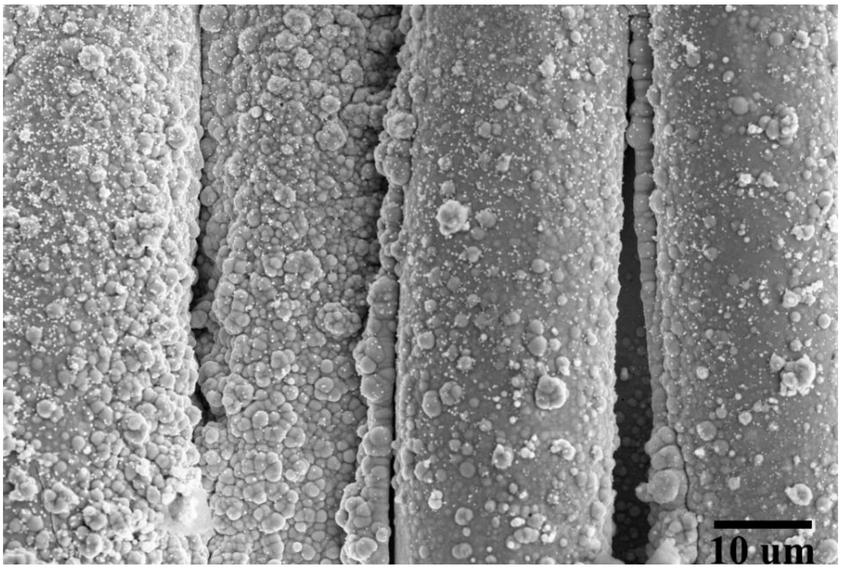

Figure S2. The SEM photograph of Ni-coated samples.

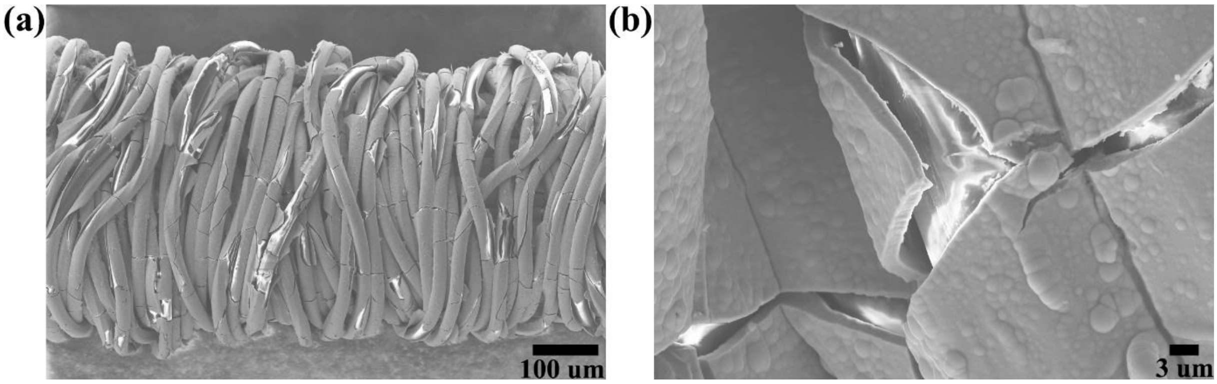

Figure S3. The SEM photograph of (a) cracked Ni-coated elastic yarns at 90 min ELD and (b) cracked Ni-coatings at high manification, showing that the thickness of Ni-layers is c.a. 1.13 µm.

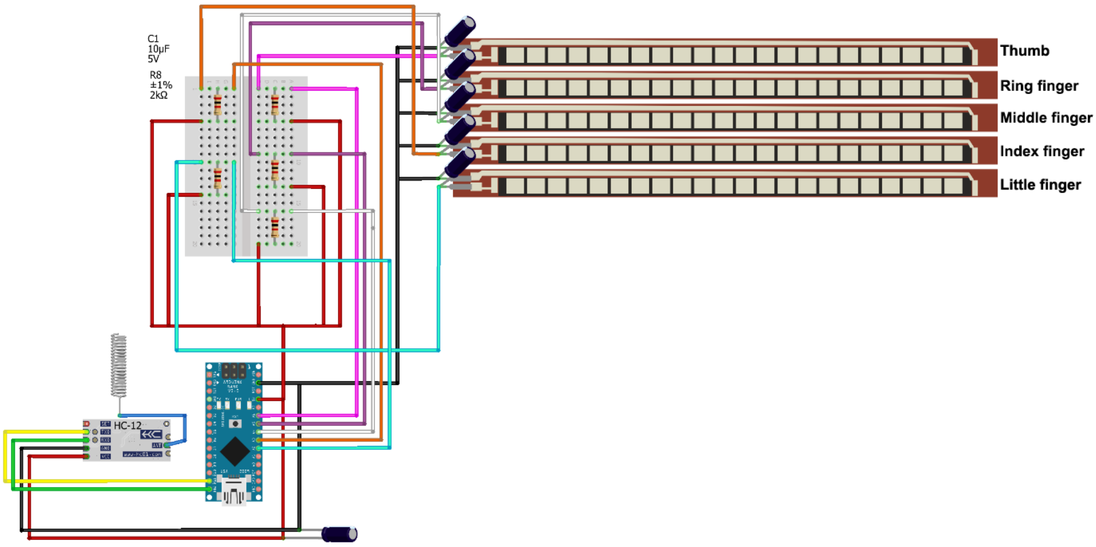

Figure S4. The overall Transmit Circuit. The 10 nF capacitor at the analog input of the Analog to Digital Converter (ADC) is needed to reduce dynamic loading effects and noises.

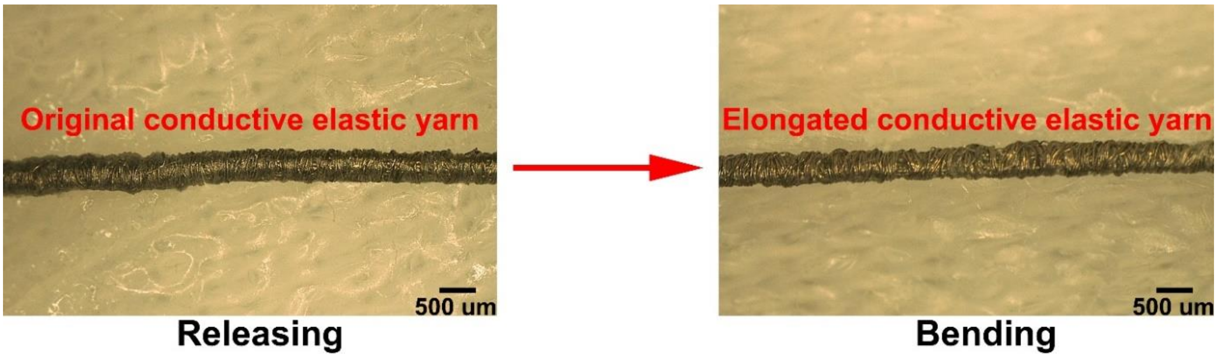

Figure S5. The microscope images of original conductive core-spun yarns (fingers releasing) and elongated Ni-coated elastic yarns (fingers bending) attached to the glove surface.

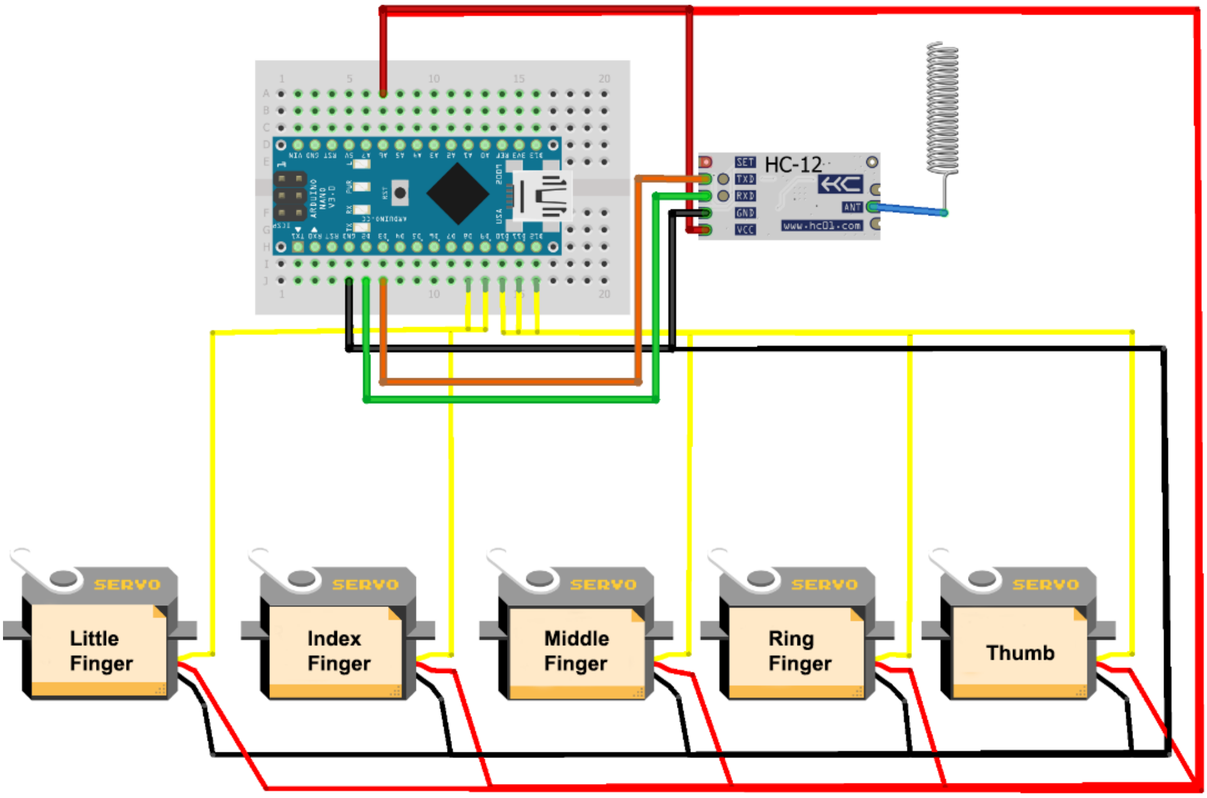

Figure S6. The overall Receive Circuit. Five Digital to Analog Converters (DACs) are used to convert digital angle value into analog angle value as input to those five servos.

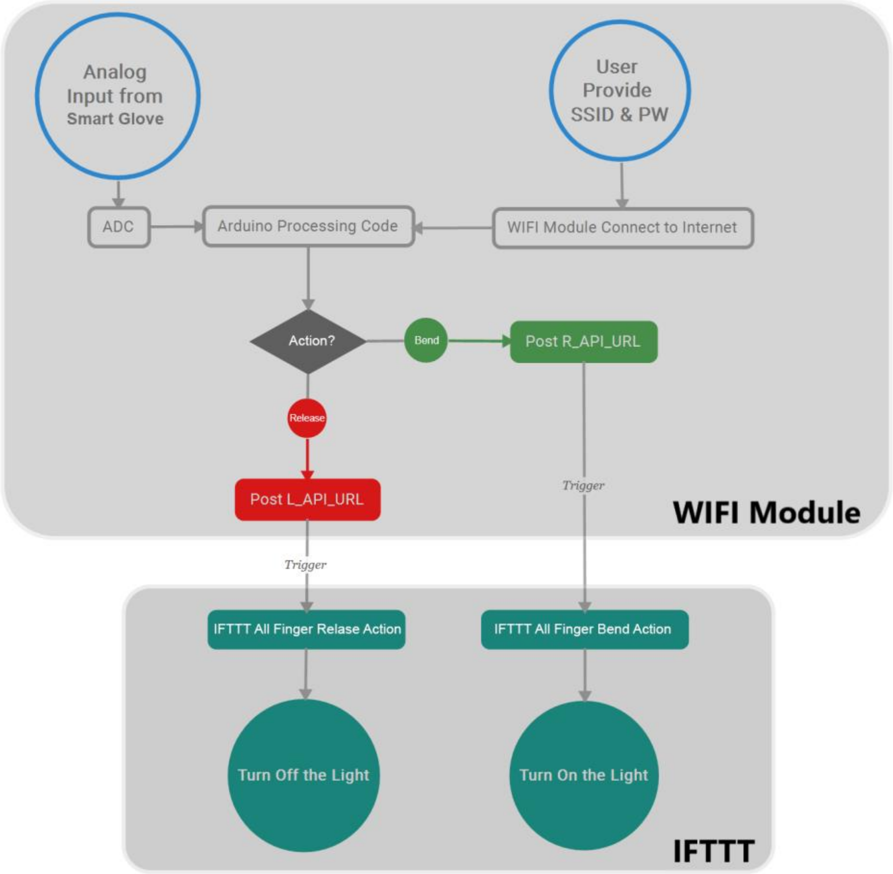

Figure S7. The designed flow chart of the If This, Then That (IFTTT) protocol based control system to turn on and off the light.

Table S1. The highest threshold and the lowest threshold of different fingers

Finger	The lowest threshold (releasing) (ohm)	The highest threshold (bending) (ohm)
Thumb	949.5	1233.4
Ring finger	1950.7	2454.4
Middle finger	2973.6	3874.0
Index finger	4515.7	5420.1
Little finger	5938.1	7260.5

Supporting Movies

Movie S1. The robotic hand accurately reflected the user’s real hand gestures.

Movie S2. As-made smart gloves changed the color of light based on different hand gestures.

Theoretical study on resistance responses of metal-coated core-spun yarns under tension and bending

In this supporting document, the detailed theoretical studies on the resistance responses of metal-coated core-spun yarns under tension and bending are presented. As shown in Figure 4a, SSSYs are composed of three parts: PU core fibers, nylon yarns wrapped around the core, and metal (Cu or Ni) film coated on the surface of nylon fibers. Therefore, the resistance R of SSSYs is equal to R_{PU}, R_{nylon} and R_{metal} in parallel. Then R can be calculated as:

$$R = \frac{1}{\frac{1}{R_{PU}}+\frac{1}{R_{nylon}}+\frac{1}{R_{metal}}} \tag{1}$$

Since raw PU and nylon are isolators, R_{PU} and R_{nylon} are much larger than that (R_{metal}) of metals (Cu, Ni), which leads to $\frac{1}{R_{PU}}$ and $\frac{1}{R_{nylon}} \ll \frac{1}{R_{metal}}$. By neglecting $\frac{1}{R_{PU}}$ and $\frac{1}{R_{nylon}}$ in equation (1), we can obtain:

$$R = R_{metal} \tag{2}$$

The resistance R_{metal} can be calculated by using the following formula:

$$R_{metal} = \rho_{metal}\frac{L_{metal}}{S_{metal}} \tag{3}$$

where ρ_{metal} is the electrical resistivity of the metal, L_{metal} is the effective length of the metal resistor and S_{metal} is effective cross-sectional area of the metal coating. Before applying the tensile force on the PU core, the adjacent metal-coated nylon yarns are in contact with each other, as illustrated in Figure 3b. Therefore, the electric current can directly pass through the metal film on the adjacent nylon fibers. Considering only one nylon winding, its effective length L_{metal} and cross-sectional area S_{metal} can be calculated as:

$$L_{metal} = \pi r_{nylon} \tag{4a}$$

$$S_{metal} = 2\pi\left(r_{PU} + 2r_{nylon}\right) \times t \approx 2\pi r_{PU} t \tag{4b}$$

where r_{nylon} is the radius of the nylon yarn, r_{PU} is the radius of the PU core, and t is the thickness of the metal film, as shown in Figure 4b. Because of $r_{nylon} \ll r_{PU}$, r_{nylon} can be neglected in equation (4b) and by introducing equation (4) into equation (3), we can obtain:

$$R_{contact\_1} = \frac{\rho_{metal}}{2t}\frac{r_{nylon}}{r_{PU}} \quad (5)$$

Equation (5) estimates the resistance of the metal film coated on the upper surface of the nylon winding at contacting state. In fact, the electric current can also pass through the metal film on the lower part, as illustrated in Figure 4b. As the conductive path of the lower metal film is in parallel to that of the upper metal coating and theoretically, they have the same resistance, the total resistance of one metal-coated nylon winding at contacting state can be estimated as:

$$R_{contact} = \frac{1}{\frac{1}{R_{contact\_1}}\times 2} = \frac{\rho_{metal}}{4t}\frac{r_{nylon}}{r_{PU}}$$

(6)

We suppose that there are N_{total} nylon windings and since all windings are in series, the total resistance R_{0_layer} can be calculated as:

$$R_{0\_layer} = N_{total} \times R_{contact} = N_{total} \times \left(\frac{\rho_{metal}}{4t}\frac{r_{nylon}}{r_{PU}}\right) \quad (7)$$

Equation (7) estimates the resistance of one single layer of metal-coated nylon yarns. We suppose that there are n layers of nylon yarns wrapped around the PU core. Because all layers are in parallel, the total resistance R_0 in the initial state (before stretching) can be estimated as:

$$R_0 = \frac{1}{\frac{1}{R_{0\_layer}}\times n} = \frac{1}{n} \times N_{total}\left(\frac{\rho_{metal}}{4t}\frac{r_{nylon}}{r_{PU}}\right)$$

(8)

When stretching the PU core to a tensile strain ε, several adjacent nylon windings would detach, as shown in Figure 3c. Considering one detached winding, the electric current would skip this detached winding and pass through the contacted windings on other adjacent layers

with smaller resistance, as depicted in Figure 4c. We suppose that R_{detach} is the resistance of one detached winding and there are N_{detach} detached windings in each layer (assuming all layers have the same number of detached windings). Therefore, the total resistance R under tension can be estimated with the aid of equations (7) and (8) as:

$$R = \frac{1}{n}\left((N_{total} - N_{detach}) \times \left(\frac{\rho_{metal}}{4t}\frac{r_{nylon}}{r_{PU}^{*}}\right) + N_{detach} \times R_{detach}\right) \qquad (9)$$

where r_{PU}^{*} is the radius of the PU core under tension, which can be calculated as:

$$r_{PU}^{*} = (1 - \nu\varepsilon)r_{PU} \qquad (10)$$

with ν being the Poisson's ratio of PU. Introducing equation (10) into equation (9), we have:

$$R = \frac{1}{n}\left((N_{total} - N_{detach}) \times \left(\frac{\rho_{metal}}{4t(1-\nu\varepsilon)}\frac{r_{nylon}}{r_{PU}}\right) + N_{detach} \times R_{detach}\right) \qquad (11)$$

Moreover, by using equations (8) and (11), we can further calculate the relative variation of the resistance $\Delta\bar{R}$ as:

$$\Delta\bar{R} = \frac{R-R_0}{R_0} = \frac{N_{detach}}{N_{total}}\left(R_{detach}\left(\frac{\rho_{metal}}{4t}\frac{r_{nylon}}{r_{PU}}\right)^{-1} - \frac{1}{1-\nu\varepsilon}\right) + \left(\frac{1}{1-\nu\varepsilon} - 1\right) \qquad (12)$$

In equation (12), ρ_{metal} (electrical resistivity of the metal) is an intrinsic material parameter; t (thickness of the metal film), r_{PU} (radius of the PU core) and r_{nylon} (radius of the nylon yarn before stretching) are the geometric parameters of the metal-coated core-spun yarns, which are supposed to be unchanged during the tension loading. The term $\frac{N_{detach}}{N_{total}}$ in equation (12) would increase by stretching the PU core to a larger strain (i.e. more nylon windings would detach), leading to the increase of the resistance with increasing strain.

In the initial state (i.e. strain of 0), the adjacent metal-coated nylon windings are in contact with each other. By supposing that the nylon windings cover the whole surface of the PU core, we can calculate the initial length $L_{PU}^{(0)}$ of the PU core as:

$$L_{PU}^{(0)} = N_{total}\frac{2r_{nylon}}{\cos\theta_0} \qquad (13)$$

where θ_0 is the initial winding angle, as shown in Figure 4a (assuming all windings have the same winding angle). When stretching the PU core, the winding angle changes to θ and N_{detach} detached windings appear with an average gap of g, as illustrated in Figure 4c. Then the length L_{PU} of the PU core after stretching can be estimated as:

$$L_{PU} = N_{total}\frac{2r_{nylon}}{cos\,\theta} + N_{detach} \times 2g$$

(14)

From equations (13) and (14), the normal tensile strain ε can be calculated as:

$$\varepsilon = \frac{L_{PU}-L_{PU}^{(0)}}{L_{PU}^{(0)}} = \left(\frac{cos\,\theta_0}{cos\,\theta} - 1\right) + \frac{cos\,\theta_0}{r_{nylon}} \times g \times \frac{N_{detach}}{N_{total}} \quad (15)$$

From equation (15), we can further derive the formula as:

$$\frac{N_{detach}}{N_{total}} = \frac{r_{nylon}/\,cos\,\theta_0}{g}\left(\varepsilon + 1 - \frac{cos\,\theta_0}{cos\,\theta}\right) \quad (16)$$

By introducing equation (16) into equation (12), we can finally obtain the strain−resistance relation under stretching as:

$$\Delta\bar{R} = \left(\frac{R_{detach}(\varepsilon)}{\frac{\rho_{metal}}{4t}\cdot\frac{r_{nylon}}{r_{PU}}} - \frac{1}{1-\nu\varepsilon}\right)\frac{r_{nylon}/\,cos\,\theta_0}{g(\varepsilon)}\left(\varepsilon + 1 - \frac{cos\,\theta_0}{cos\,\theta(\varepsilon)}\right) + \left(\frac{1}{1-\nu\varepsilon} - 1\right) \quad (17)$$

In general cases, the winding angle θ, the average gap g of the detached nylon windings, and the resistance R_{detach} of detached winding will increase with the tensile strain ε. Therefore, before obtaining the analytical expression of $\Delta\bar{R}$ as a function of ε, $\theta(\varepsilon)$ (θ as a function of ε), $g(\varepsilon)$ (g as a function of ε), and $R_{detach}(\varepsilon)$ (R_{detach} as a function of ε) need to be determined first. For the core-spun yarns studied here, we found that the winding angle only changes a little when the recoverable strain range is below 50%, as shown in Figures 3b and 3c. By taking $\theta(\varepsilon) = \theta_0$, equation (17) can be simplified as:

$$\Delta\bar{R} = \left(\frac{R_{detach}(\varepsilon)}{\frac{\rho_{metal}}{4t}\cdot\frac{r_{nylon}}{r_{PU}}} - \frac{1}{1-\nu\varepsilon}\right)\frac{r_{nylon}/\,cos\,\theta_0}{g(\varepsilon)}\varepsilon + \left(\frac{1}{1-\nu\varepsilon} - 1\right) \quad (18)$$

Considering the simplest case, where $g(\varepsilon)$ is constant (=g_0) and $R_{detach}(\varepsilon)$ is also constant (= $R_d^{(0)}$), equation (18) can be further simplified as:

$$\Delta\bar{R} = \left(\left(\frac{R_d^{(0)}}{\frac{\rho_{metal}}{4t}\frac{r_{nylon}}{r_{PU}}} - \frac{1}{1-\nu\varepsilon}\right)\frac{r_{nylon}/\cos\theta_0}{g_0}\right) \times \varepsilon + \left(\frac{1}{1-\nu\varepsilon} - 1\right) \quad (19)$$

From the perspective of bending behaviour in the smart glove system, the whole heterogeneous structure can be simplified as a beam (substrate) with metal-coated core-spun yarn (functional component SSSYs) attached to its top surface, as shown in Figure 4e. When bending the substrate beam, the top surface of the beam would elongate, causing the tensile force applied on the SSSYs. The resulted tensile strain will lead to an increase in the resistance of the core-spun yarns due to the detached windings (see Figure 3c), and an analytical relation between the tensile strain and the relative variation of resistance has already been derived (equation (19)).

Under the assumption of pure bending, the tensile strain ε of SSSYs can be estimated as:

$$\varepsilon = \frac{c}{\rho} \quad (20)$$

where c is the distance between the top surface of the beam and its neutral surface (i.e. the surface at which the tensile strain is 0%), and ρ is the radius of the deformed beam (see Figure 4e). The bending angle α is related to the curvature $\frac{1}{\rho}$ of the beam by the following formula:

$$\alpha = \frac{L}{\rho}$$

(21)

where L is the initial length of the beam. With the aid of equations (19-21), we can obtain the bending angle–resistance relation as the following formula:

$$\Delta\bar{R} = \left(\left(\frac{R_d^{(0)}}{\frac{\rho_{metal}}{4t}\frac{r_{nylon}}{r_{PU}}} - \frac{1}{1-\nu\cdot\frac{c}{L}\cdot\alpha}\right)\frac{r_{nylon}/\cos\theta_0}{g_0}\frac{c}{L}\right) \times \alpha + \left(\frac{1}{1-\nu\cdot\frac{c}{L}\cdot\alpha} - 1\right) \quad (22)$$
